# Molecular Mechanisms of Pregnancy-Related Vascular Remodeling and Pregnancy Complications

**DOI:** 10.3390/ijms24043712

**Published:** 2023-02-13

**Authors:** David K. Stevenson, Ronald J. Wong, Nihar R. Nayak

**Affiliations:** 1 Department of Pediatrics, Division of Neonatal and Developmental Medicine, Stanford University School of Medicine, Stanford, CA 94305-1509, USA; 2 Department of Obstetrics and Gynecology, UMKC School of Medicine, Kansas City, MO 64108, USA

The purpose of this editorial is to highlight the various observations made in this Special Issue in the International Journal of Molecular Sciences. Most of the “great obstetrical syndromes” such as pre-eclampsia, preterm birth, and intrauterine growth restriction (IUGR) are characterized by abnormalities in placental vascular development. However, the molecular mechanisms contributing to these developmental deviations are less well characterized, as they involve multiple cell types and the evolution of their roles throughout gestation, including specific signaling behaviors and pathways. Indeed, they involve the disruption of normal development during pregnancy; the intricate immunological harmony between the mother and the fetus is orchestrated by the placental cells at the center of these syndromes. Moreover, their occurrences may have implications for the long-term health of both the mother and infant, such as later development of hypertension and cardiovascular disease (CVD), suggesting persistent abnormalities in cell signaling that may be long lived.

One possible mechanism for impaired placental vascular development is described in the article by Yoshida et al. in 2021 [1], which reports on the role of alpha-1 antitrypsin (A1AT) in placental vascular development. The results suggest that the failure to upregulate A1AT expression could cause defective extravillous trophoblast (EVT) invasion, leading to impaired vascular remodeling of the spiral arteries (SAs) and hypertension or pre-eclampsia. The effects seem to be mediated through “high-temperature requirement A serine peptidase 1” (HTRA1) expression in the EVT cells and endoplasmic reticulum (ER) stress. Nonetheless, a full characterization of the mechanism remains to be elucidated, particularly regarding the failure to upregulate A1AT in response to factors such as an ischemic placenta, the dysregulation of angiogenesis, and the presence of excessive oxidative stress.

Using a murine model of perinatal infection (endotoxic lipopolysaccharide (LPS)-induced inflammation), in 2021, Panja et al. [2] administered LPS to pregnant mice at the time of blastocyst implantation, which is a naturally occurring pro-inflammatory event provoked by the semi-allogeneic blastocyst, to distinguish between the natural event and the infectious circumstance. Their data suggest that neutrophils are not involved in the MyD88 pathway inflammatory cascade initiated by LPS induction. Moreover, the natural event seems to involve immune cell movement away from the early blastocyst implantation site (EBIS) and immune cell signaling that regulates the inflammatory response, protecting the blastocyst from the kind of response associated with perinatal infection and preventing early fetal loss and/or other pregnancy complications.

In 2021, Hu and Zhang [3] reviewed the functional adaptation of the uteroplacental circulation in normal pregnancy and pre-eclampsia based on relevant papers using human and animal models published over the last several decades. By acknowledging that SA remodeling contributes importantly to the hemodynamic adaptation in normal pregnancy and that defective remodeling contributes to the maladaptation observed in pre-eclampsia, the article simply describes the various factors that differ in the two situations in terms of vascular tone, myogenic tone, metabolic influences, neural influences, shear stress, and vasoconstrictors and vasodilators. Various mechanisms are hypothesized for these changes, but, they did not address the fundamental issue of what triggers the failure in placental vascular adaptation. Unfortunately, the pathophysiology that has been described in the literature to date is simply a derivative of the observation that an initial trigger followed by a system-wide series of maladaptive signaling ultimately leads to the evolution of the various pathologic phenotypes.

In 2021, Naicker et al. [4] attempted to make the case for a synergy between pre-eclampsia, human immunodeficiency virus (HIV), and antiretroviral therapy (ART) in terms of oxidative stress and endothelial dysfunction. Thus, not surprisingly, HIV and its therapy might contribute to the severity and lethality of pre-eclampsia. Nonetheless, oxidative stress is not always bad, especially in the context of hypoxia. Indeed, oxygen levels in the placental microenvironment vary throughout gestation, with severe hypoxic conditions characterizing the first trimester, a critical time for placental development, including blastocyst implantation, cytotrophoblast invasion, and SA remodeling initiation. In 2021, Zhao et al. [5] drew this conclusion: nature strikes the correct balance between oxidation and anti-oxidation at the ideal times to achieve successful gestation in most pregnancies. We are reminded that the tissue oxygen level is a powerful regulator of cellular signaling behavior and crosstalk between different cell types. A defect at any point in the developmental process can lead to a subsequent cascade of metabolic consequences, resulting in differing phenotypes depending on the timing of the insult and cell types involved. To this extent, it can be understood that preterm birth, pre-eclampsia, and intrauterine growth restriction may lie on the same developmental spectrum, despite their very different clinical presentations. Of course, abnormal placental vascular development remains a unifying theme in all three phenotypes. In 2021, Halari et al. [6] considered the cause of pregnancy-associated diseases by introducing the role of two proteoglycans, biglycan and decorin, in these same processes.

In 2021, Munjas et al. [7] reminded us that non-coding RNAs, particularly microRNAs (miRNAs) and long non-coding RNAs (lncRNAs), may have a role in the pathophysiological processes leading to pre-eclampsia. Because they can be detected in the maternal circulation during gestation, they could serve as biomarkers of pre-eclampsia, although lncRNAs are present in lower abundance, requiring special sampling and analytic techniques. This is not surprising, as circulating cfRNA has been used to predict preterm birth and preterm birth early in gestation, long before the onset of preterm labor or any clinical signs of pre-eclampsia [8,9].

In 2021, Hong et al. [10] speculated as to the causation of the well-recognized link of pre-eclampsia, and later, CVD in women. Once again, inflammatory signaling is at the core of the pathogenesis. However, it remains uncertain whether certain women are prone to developing CVD prior to becoming pregnant, manifesting their propensity for such hemodynamic dysfunction during gestation, or if they develop hemodynamic dysfunction because of defects in the placentation process and early placental vascular development that somehow permanently alter their risk profile for CVD. Large studies are underway to resolve this quandary, as well as predict who among the pregnant women are at risk for pre-eclampsia, and later, CVD.

In 2022, Shukla and Soares [11] studied trophoblast invasion and SA remodeling using a rat model, while acknowledging the differences between the rat and human placentas. Despite the limitations, which the authors make a case for focusing on trophoblasts, a chameleon cell type, working at the interface between the fetus and the mammalian mother to ensure that the maternal environment is a hospitable one for the implantation of a semi-allogeneic blastocyst, and subsequently, normal placental development.

In conclusion, this Special Issue is a potpourri of investigative efforts and perspectives on two of the most profound events in mammalian nature: allogeneic blastocyst implantation into the uterus and the cohabitation of two distinct individuals for a specified maturational timeframe to prepare the fetus for a totally independent existence. Solving this biological mystery of the highly regulated tolerance, and then rejection of the fetus at the molecular level will likely provide insights into not only normal parturition, preterm birth, pre-eclampsia, and IUGR, but also into cancer, autoimmunity, and CVD, and maybe even aging. All of these pathologic conditions reflect dysregulated or ill-timed inflammatory responses. Natural pregnancy makes use of inflammatory processes at the correct right places, doses, and times. We have only to learn the essential information, and then invent methods to solve the inflammatory problems that plague our species.
